# Phase Transformation Temperature Prediction in Steels via Machine Learning

**DOI:** 10.3390/ma17051117

**Published:** 2024-02-29

**Authors:** Yupeng Zhang, Lin Cheng, Aonan Pan, Chengyang Hu, Kaiming Wu

**Affiliations:** The State Key Laboratory of Refractories and Metallurgy, Hubei Province Key Laboratory of Systems Science on Metallurgical Processing, International Research Institute for Steel Technology, Collaborative Center on Advanced Steels, Wuhan University of Science and Technology, Wuhan 430081, China; zhangyupeng2024@163.com (Y.Z.); 18055678079@163.com (A.P.);

**Keywords:** phase transformation temperature, steels, machine learning, atomic parameter

## Abstract

The phase transformation temperature plays an important role in the design, production and heat treatment process of steels. In the present work, an improved version of the gradient-boosting method LightGBM has been utilized to study the influencing factors of the four phase transformation temperatures, namely Ac1, Ac3, the martensite transformation start (MS) temperature and the bainitic transformation start (BS) temperature. The effects of the alloying element were discussed in detail by comparing their influencing mechanisms on different phase transformation temperatures. The training accuracy was significantly improved by further introducing appropriate features related to atomic parameters. The melting temperature and coefficient of linear thermal expansion of the pure metals corresponding to the alloying elements, atomic Waber–Cromer pseudopotential radii and valence electron number were the top four among the eighteen atomic parameters used to improve the trained model performance. The training and prediction processes were analyzed using a partial dependence plot (PDP) and Shapley additive explanation (SHAP) methods to reveal the relationships between the features and phase transformation temperature.

## 1. Introduction

The microstructure and mechanical properties of steels depend on chemical composition, plastic deformation and heat treatment process [1,2,3,4,5,6]. Phase transformation start and finish temperatures in steels, marking the initial formation of the diverse microstructure, are crucial in designing steels with different targeted microstructures [7,8], such as martensite and bainite, as well as other advanced high-strength steels with complex microstructures, for example, medium manganese steels requiring inter-critical annealing [9,10,11,12]. The martensite transformation start (MS) temperature has attracted significant interest over the years because lath martensite is a base microstructure constituent for most high-strength steels [13]. Numerous methodologies, such as thermodynamics-based methods, linear regression, artificial neural network (ANN) modeling and machine learning, have been applied to predict the martensite transformation start (MS) temperature [14,15,16]. In the meantime, due to the virtues of bainitic steels, such as high strength, ductility, toughness and creep resistance at reasonable costs, the austenite-to-bainite transformation has also gained lots of interest [17]. Ac1 and Ac3 temperatures are the main parameters used to design the heat treatment process of steels. For example, medium Mn steel (3–12 wt. Mn%), as one of the various candidate steels for third-generation AHSS, typically consists of an ultrafine-grained dual-phase (austenite–ferrite) microstructure obtained through the inter-critical annealing (annealing between Ac1 and Ac3 temperature) of the quenched martensite matrix [18]. However, studies on the prediction of the BS temperature [19] and Ac1 and Ac3 temperatures [20] by means of machine learning are limited compared with those on the prediction of the MS temperature. Systematic and comparative studies on the influencing factors of the main phase transformation temperature in steels have not been reported. Meanwhile, most research has focused on the accuracy of trained models, but the focus on the training process and explanation of the trained models has been limited. This work aims to conduct in-depth research on these two concerns.

The main drawback of the empirical formulation is that it utilizes a linear equation to describe the relationship between the alloying element content and the phase transformation start temperature [21,22,23]. The general structure of the equation is as follows [24]:

(1) $$F\left(X\right)={\left[w^{1},w^{2},w^{3}\cdots w^{N}\right]\times \left[k^{1},k^{2},k^{3}\cdots k^{N}\right]}^{T}+k_{0}$$

where $k_{1}k_{2}k_{3}\cdots k_{N}$ represent the content of alloying elements (wt%). Similarly, $w_{1}w_{2}w_{3}\cdots w_{N}$ denote the weight coefficients, and $k_{0}$ is the bias coefficient. Normally, empirical formulas are only applicable to limited steels. For machine learning, early on, the chemical composition was chosen as the input feature to train models [25]. Recently, new features related to a simplified but still complicated Gibbs energy change description have been included in the feature space to improve the performance of trained modes, and significant improvements have been achieved. Moreover, recently, to predict the martensite transformation start temperature in Fe–C–X alloys, researchers constructed a complicated formula to represent the main part of the non-driving force of martensite transformation [26]. However, in our previous work [27], it was found that the addition of new features related to six atomic parameters significantly improved the performance of the trained model to predict the martensite transformation temperature. Therefore, in this work, instead of constructing complicated formulas to describe the driving force and/or resistance force of the specific phase transformation, more atomic parameters were considered to construct new features.

To improve the performance of the trained prediction model of the phase transformation temperature, a lot of efforts have been made, such as parameter tuning, principal component analysis, careful training dataset preparation, diverse dataset cleaning methodology, different machine learning method comparisons [28] and so on. Wang et al. [29] integrated deep data mining of thermodynamic calculations with a deep learning framework to develop a versatile and scalable model for the prediction of the martensite transformation start temperature. Thermodynamic calculations enhance the information in a feature set but necessitate specialized computational software and databases. Lu et al. [30] utilized thermodynamic knowledge in combination with a multi-layer feedforward neural network to reduce the dimension of the feature space through kernel principal component analysis. Furthermore, a genetic algorithm was employed to find the appropriate hyperparameters to predict the martensite transformation start temperature of steels. Peet et al. [31] utilized a combination of a thermodynamic model and a Bayesian neural network to predict the martensite transformation start temperature. Tian et al. [32] assessed four machine learning models, namely random forest regression, support vector regression, linear regression and XGB regression. Both random forest and XGB, which are based on tree models, demonstrate excellent performance, suggesting that integrated algorithms coupled with tree models are effective in addressing nonlinear problems. Most existing machine learning models struggle with interpretability. Tree-based models, including random forest and gradient-boosting tree models, exhibited natural and strong interpretability. These integrated models not only retain the excellent interpretability of tree models but also offer superior performance, addressing the limitations associated with interpretability in traditional models. LightGBM is an improved and high-performance gradient-boosting framework with higher efficiency and accuracy [33]. For example, recently, LightGBM outperformed other classic machine learning methods, such as XGBoost, random forest, SVR and Lasso, in the prediction of the corrosion rate of 3C steel [34], and other boosting methods, such as adaptive boosting (AdaBoost), gradient-boosting machine (GBM), extreme gradient boosting (XGBoost) and categorical gradient boosting (CatBoost), in the prediction of the sequence of plastic hinge formation in steel frame structures [35].

Therefore, in this work, to deepen the understanding of the influence mechanisms of alloying elements on phase transformation temperatures, a systematical study of the four transformation temperatures was conducted. LightGBM was chosen as the machine learning algorithm integrating atomic parameter descriptors. Furthermore, to improve the understanding of the prediction models, the partial dependence plot (PDP) and Shapley additive explanations (SHAP) analysis methods were utilized to explain the trained models and the prediction results.

## 2. Methodology

### 2.1. LightGBM Algorithm

In the present work, an improved version of the gradient-boosting method LightGBM was chosen as the machine learning algorithm [36]. LightGBM yields better training speed and prediction accuracy by using improved measures such as histogram-based algorithms, which bucket the continuous values of features into discrete bins, and a leaf-wise tree growth strategy wherein the leaf with maximum loss is selected to grow and, therefore, the number of leaves at each level is not always the same [37,38].

### 2.2. Evaluation Metrics

Mean absolute error (*MAE*) and the coefficients of determination (*R*^2^) were utilized to evaluate the accuracy of the trained model. Mean absolute error (*MAE*), representing the mean absolute error between the predicted value and the real value, can be expressed as follows:

(2) $$MAE=\frac{1}{N}\sum_{i=1}^{N}\left|y_{i}-Y_{i}\right|$$

where N represents the number of the samples, and $y_{i}$ and $Y_{i}$ represent the real and predicted values of the ith sample, respectively. The smaller the MAE is, the better the performance [39].

The coefficient of determination (*R*^2^) indicates the amount of dependent variable Y that can be accounted for by the independent variable x in the regression model, expressed as follows:

(3) $$R^{2}=1-\frac{\sum_{i=1}^{N}{\left(Y_{i}-y_{i}\right)}^{2}}{\sum_{i=1}^{N}{\left(Y_{i}-\bar{y}\right)}^{2}}$$

where N refers to the number of samples. $y_{i}$, $Y_{i}$ and $\bar{y}$ represent the real value of the ith sample, the predicted value of the ith sample and the average of real values, respectively. The larger *R*^2^ is, the better the performance [40].

### 2.3. Model Interpretability Metrics

Interpretability is crucial in understanding the trained models by means of machine learning methods [41]. In this work, PDP and SHAP were used to explore the relationships between the features and phase transformation temperatures. Partial dependence plots (PDP) describe the marginal influence of one or two features on the prediction outcome of a trained machine learning model:

(4) $$\hat{f}\left(x_{s}\right)=E_{x_{c}}\left[\hat{f}\left(x_{s},x_{c}\right)\right]=\int f(x_{s},x_{c})dP(x_{c})$$

where $x_{s}$ is the set of the features for which the partial dependence function should be plotted and $x_{c}$ represents the other remained features utilized in the machine learning model. Features $x_{s}$ and the set $x_{c}$ together make up the total feature space. By marginalizing over the features in set $x_{c}$, a function that depends on the features in $x_{s}$ is then obtained [42]. Shapley additive explanations (SHAP) comprise an interpretative method of machine learning based on game theory. The formula is as follows:

(5) $$g\left(Z^{’}\right)=\varphi_{0}+\sum_{i=1}^{M}\varphi_{i}{Z_{i}}^{’}$$

where $Z^{’}\in {\left\{0,1\right\}}^{M}$, M is the number of input features, and $\varphi_{i}\in R$. The ${Z_{i}}^{’}$ variables typically represent a feature being observed $\left({Z_{i}}^{’}=1\right)$ or unknown $\left({Z_{i}}^{’}=0\right)$, and the ${\varphi_{i}}^{’}s$ are the feature attribution values [43,44,45].

### 2.4. Machine Learning Strategy

**Figure 1:**
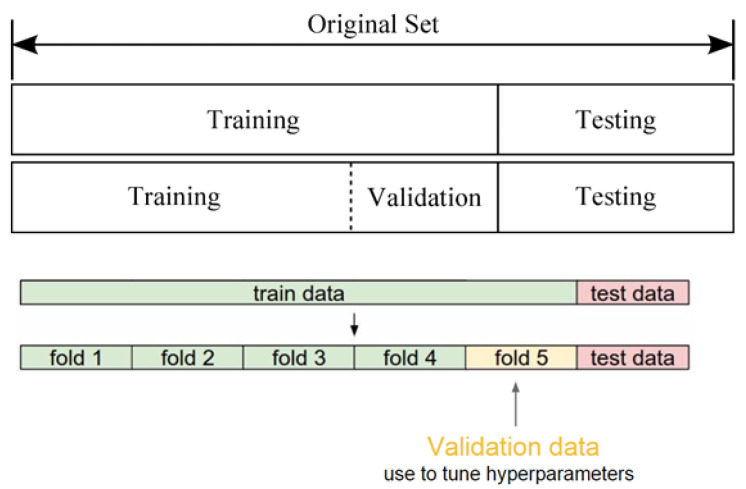
K-fold cross-validation process.

**Figure 2:**
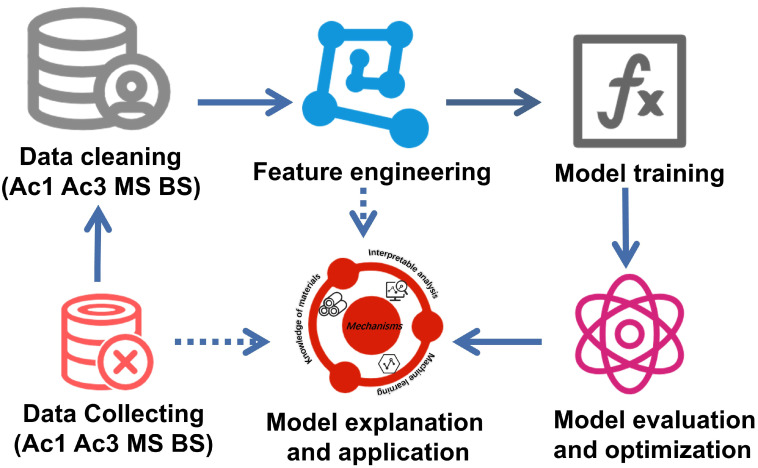
The workflow of the present work.

K-fold cross-validation was utilized in the present work as presented in Figure 1. The dataset was subdivided into K subsets which were independent of each other. Each subset was selected as a test set in turn, and the remaining K − 1 subsets were selected as a training set. The performance of the selected machine learning method was evaluated by obtaining the averaged prediction accuracies of the K tests [46]. The workflow of the present work is shown in Figure 2.

## 3. Results

### 3.1. Data Collection and Screened

The original dataset of the present work was downloaded from a subset of the phase transformation database named the Materials Algorithms Project (MAP), provided by University of Cambridge. Although the Materials Algorithm Project (MAP) is an open scientific research project, it originated from a high-quality joint project of the University of Cambridge and the National Physical Laboratory and was sponsored for four years by the Engineering and Physical Sciences Research Council (EPSRC) of the United Kingdom [47,48,49]. To ensure data quality, entries with the same chemical composition but with different MS values were removed. In both Ac1 and Ac3 datasets, terms with the same chemical composition and heating rate but with different Ac1 and Ac3 values were removed. In the BS dataset, terms with the same chemical composition and cooling rate but with different BS values were removed. The sizes of the four datasets after data cleaning are shown in Table 1, and the numbers of deleted samples in each dataset are given in Table 2. The overall information of MS, Ac1 and Ac3, and BS datasets are shown in Table 3, Table 4 and Table 5, respectively.

Atomic parameters could be divided into two categories. One was associated with the properties of the free atoms, such as radius, electronegativity and the ionization energy of the atoms, etc. The other was related to the pure metals corresponding to the alloying elements [50]. The atomic parameters utilized in the present work are shown in Table 6.

### 3.2. Performance of Empirical Formula

**Figure 3:**
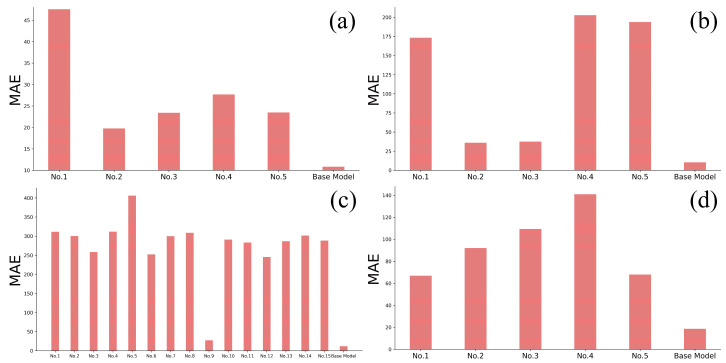
Comparison between the performance of the empirical formulations and trained machine learning models without atomic parameters. (**a**) Ac1, (**b**) Ac3, (**c**) MS and (**d**) BS.

The collected empirical formulas sued to predict Ac1 and Ac3 temperatures, the bainite transformation start temperature (BS temperature) and the martensite transformation start temperature (MS temperature) are shown in Table 7, Table 8, Table 9 and Table 10 [20,21,22,24,25,50,51,52,53,54,55,56,57,58], respectively. The performance of the empirical formulas was evaluated on the dataset provided by the Materials Algorithms Project (MAP) and compared with four preliminary machine learning models (called base models) trained using the same datasets. Four base models using only the chemical compositions, the cooling or heating rates, and the corresponding phase transformation temperature were trained based on the MAP dataset. During training, n_estimators was set as 600, random_state was set as 8 and all other hyperparameters in LightGBM were used with default values. The performances of the four trained models and the empirical formulas were compared on the remaining part of MAP dataset, which was not utilized in the training of the models. Feature sets of the machine learning models are shown in Table 11. It is clear that the empirical formulas exhibit larger errors compared with the models trained by means of machine learning (as shown in Figure 3), which indicates that the empirical formulas exhibit inherent limitations and deficiencies, thereby restricting their applicability [59,60].

### 3.3. Performance of the Machine Learning Models Trained with Atomic Parameters

**Figure 4:**
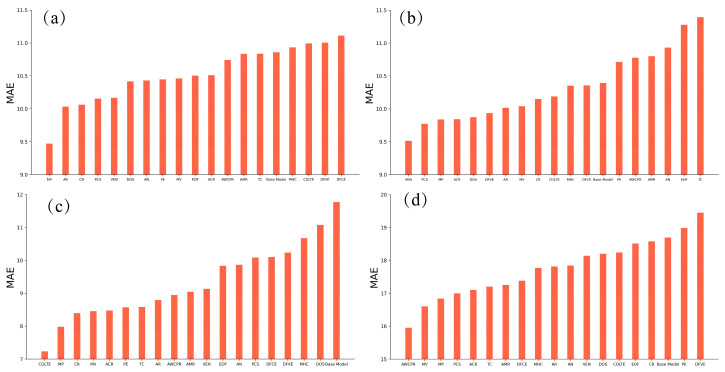
MAE of the trained models by machine learning with atomic parameters. (**a**) Ac1, (**b**) Ac3, (**c**) MS and (**d**) BS.

**Figure 5:**
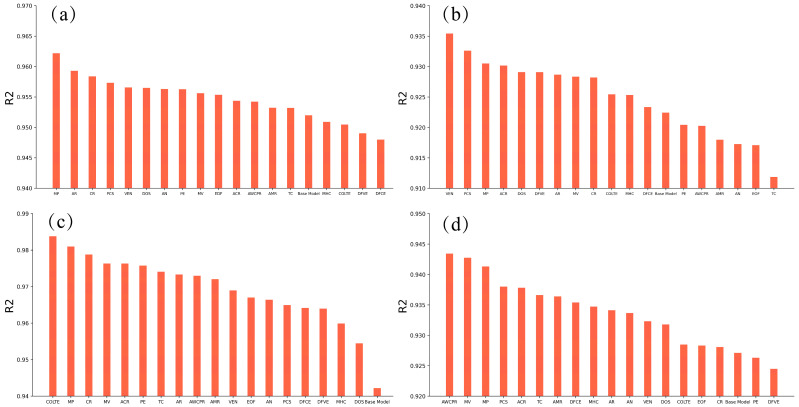
R2 of the trained models by machine learning with atomic parameters. (**a**) Ac1, (**b**) Ac3, (**c**) MS and (**d**) BS.

**Figure 6:**
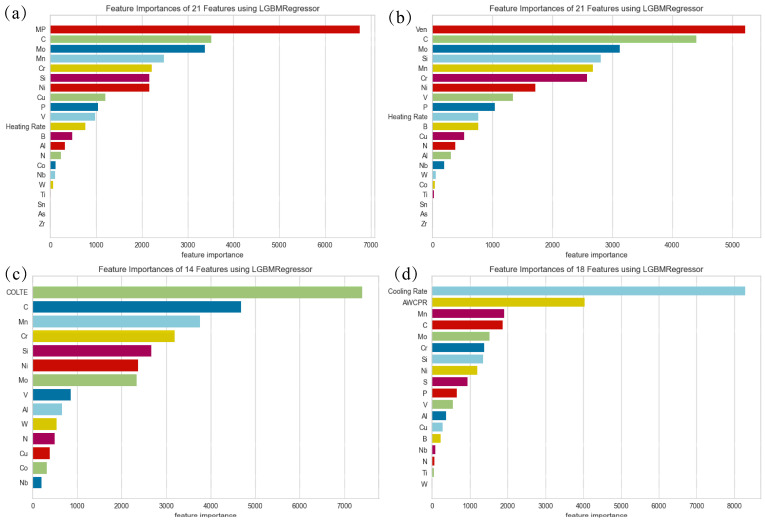
Feature importance analysis in the training process. (**a**) Ac1, (**b**) Ac3, (**c**) MS and (**d**) BS.

In our previous work [27], it was found that the introduction of new features related to atomic parameters could significantly improve the performance of trained models in predicting the martensite transformation start temperature. In this work, more complete atomic parameters (18 types) were introduced to construct new features. Each atomic parameter was separately introduced into the feature space to train a new model and then comparisons among the 19 models were conducted as shown in Figure 4 and Figure 5 under different evaluation indexes. Only the atomic parameters were attached in each sample and the size of the dataset was not changed. Meanwhile, the models with atomic parameters were trained with the same hyperparameters as the base models. The newly trained model was named by the abbreviation of the introduced atomic parameter as shown in Table 6. The model without any atomic parameter was named the base model. It was found that all the models used to predict the martensite start transformation temperature with atomic parameters outperform the base model. For the other three phase transformation temperatures, most of the models with atomic parameters outperform the base model. Specifically, the melting temperature and linear thermal expansion coefficient of the pure metal related to the alloy element, the valence electron number and pseudopotential radius come first in the ranking of the most effective atomic parameters in training Ac1, MS, Ac3 and BS prediction models, respectively. In Figure 6, it is shown that except for the BS prediction model, the newly introduced feature related to the atomic parameters ranks first regarding the importance of the features in the other three models.

**Figure 7:**
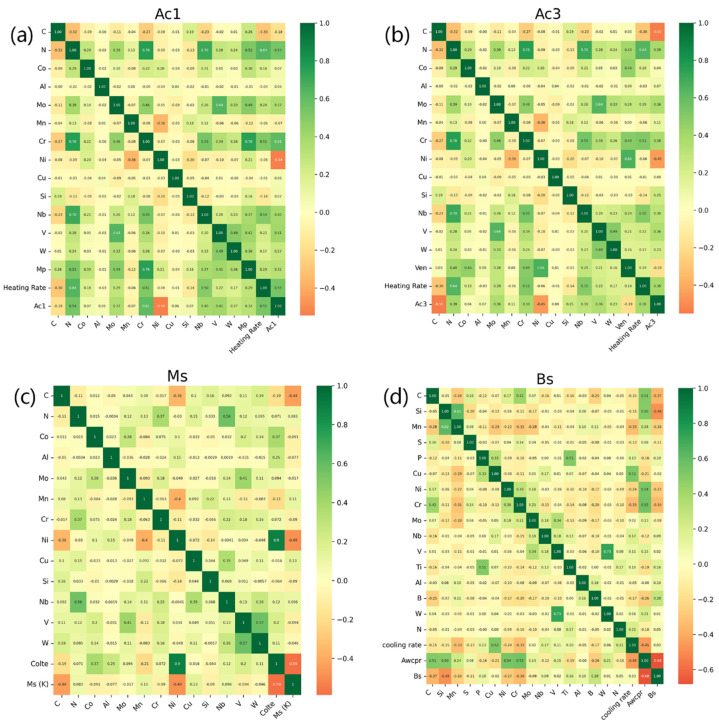
Feature correlation heatmap between the features and the specific phase transformation temperature. (**a**) Ac1, (**b**) Ac3, (**c**) MS and (**d**) BS.

**Figure 8:**
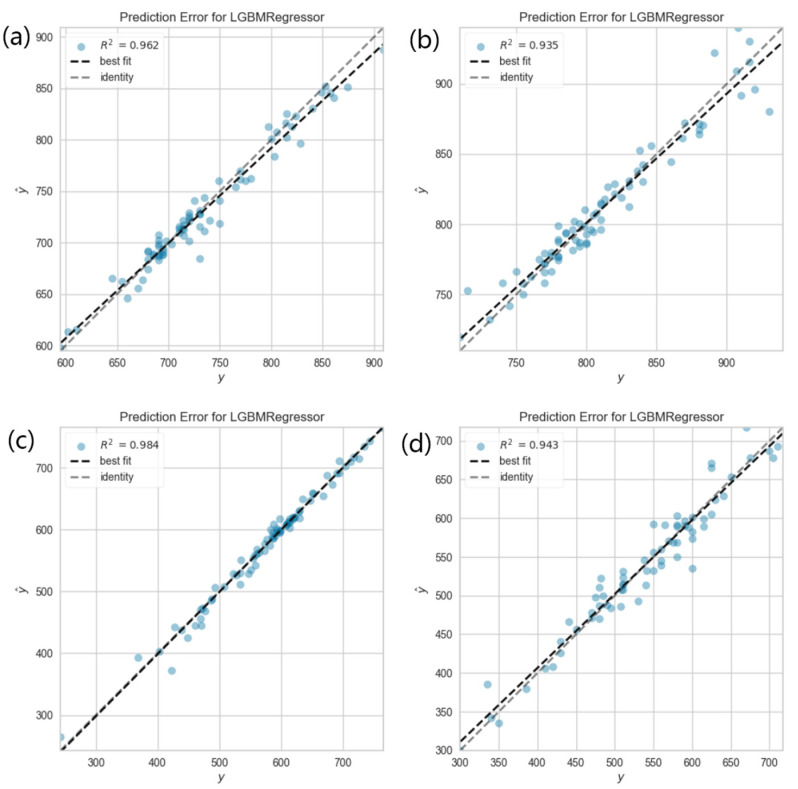
The fitting results in the models with the best atomic parameters. (**a**) Ac1, (**b**) Ac3, (**c**) MS and (**d**) BS.

In Figure 7, it is shown that Pearson’s linear correlation coefficient between any two features in the new feature space with the addition of best atomic parameters was below 0.7 for four models, suggesting a limited correlation between the features and no extra feature screening was needed. Figure 8 exhibits the final trained models with the best atomic parameters. It is clear that all the points are close to the diagonal lines, which indicates that these four models were trained with high accuracy. Table 12 and Table 13 show the evaluation results of the trained models with and without adding features related to atomic parameters, respectively. It is clear that the fitting error was significantly decreased. The MAE is decreased by 1.604 °C, 0.932 °C, 4.785 °C and 2.659 °C for Ac1, Ac3, MS and BS, respectively. The R2 is increased by 0.010, 0.019, 0.042 and 0.015 for Ac1, Ac3, MS and BS, respectively. The evaluation indexes are systematically improved. Especially, the performance of the trained MS prediction model was significantly increased.

## 4. Discussion

### 4.1. The Influencing Factors of MS Temperature

The nucleation and growth rate of the new phase were closely tied to the chemical composition of the steels during the solid phase transformation [61,62,63]. Table 14 shows the clarification of normal alloying element in the steels. Generally, the austenite-forming elements should stabilize the austenite during the cooling stage and prompt its transformation during the heating stage. Vice versa, the ferrite-forming elements should stabilize the ferrite during the heating stage and prompt its transformation during the cooling stage. Meanwhile, the effects of carbon on the phase transformation could be influenced differently by carbide-forming elements and non-carbide-forming elements.

It is generally accepted that all alloying elements except Al and Co lower the MS temperature. Different from martensite transformation [27], the influencing mechanisms of the alloying elements on MS temperature were explained in detail in our previous publication [27]. In summary, the higher the C, Ni, Cr, Mn and Si contents, the lower the SHAP values (negative), i.e., the lower the MS temperature; the higher the Al, Co and V contents, the higher the SHAP values (positive), i.e., the higher the MS temperature; N, Nb and Cu show a similar effect on MS temperature as Al, Co and V do but with some exceptions. W and Mo demonstrate more complicated effects on MS temperature.

Among all the alloying elements, C exhibited the most pronounced effects on austenite decomposition temperature with decreasing ability at higher contents. Mn and Ni were negatively related to the MS temperature, consistent with the thermodynamic mechanism. The influence of alloying elements on the MS temperature was primarily governed by their impact on the T_0_ temperature at which the ferrite and austenite with the same chemical composition showed equal free energy and their ability in strengthening the prior austenite phase. C demonstrated a significant effect in strengthening the austenite phase and demonstrated a substantial reduction in the MS temperature. Likewise, the presence of Mn, Ni, Cu and some other austenite-forming elements was associated with a decrease in the T_0_ and a marginal effect in strengthening the austenite, leading to a significant decrease in the MS temperature. On the other hand, ferrite-forming elements such as Al, Co, Si, Mo, W, V and Ti were found to elevate the T_0_ but still enhanced the strength of prior austenite to different extents, as indicated by various studies [64,65,66,67,68,69]. Therefore, mostly, the addition of alloying element inhibits the formation of martensite and, correspondingly, decreases the MS temperature.

**Figure 9:**
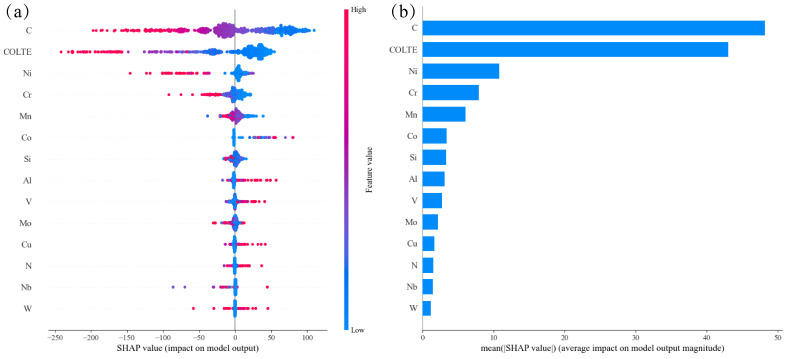
SHAP analysis in the trained MS prediction model: (**a**) impact on model output and (**b**) average impact on model output magnitude.

Figure 9 shows the importance of the features in the model with the best performance after adding a new feature, the coefficient of linear thermal expansion of the pure metals corresponding to specific alloying elements. In Figure 6, it is shown that this feature ranked first in importance in fitting the machine learning model, and Figure 9 further demonstrates that this feature significantly contributed to the prediction outcome, second only to C, which highlighted the importance of new feature construction. In Figure 7, it is shown that COLTE showed the largest positive correlation coefficient with Ni and the second and third largest positive correlation coefficients with Co and Al, respectively. In the meantime, both C and Mn exhibit negative and close correlation coefficients with COLTE. Further, as shown in Figure 9, the higher the COLTE values, the lower the MS temperature. These results revealed that the alloying elements demonstrate complicated effects on the MS temperature because they contribute to both sides, the driving force and the resistance force, i.e., non-chemical driving forces.

The non-chemical driving forces ($\Delta G^{a\to M}$) in the martensitic phase transformation include the dilatation strain energy ($\Delta G_{dil}$), dislocation stored energy ($\Delta G_{stor}$), the shearing energy of austenite ($\Delta G_{sh}$) and interfacial energy ($\Delta G_{inter}$), which can be specified as in Equation (6) [68],

(6) $$\Delta G^{a\to M}=\Delta G_{sh}+\Delta G_{dil}+\Delta G_{stor}+\Delta G_{inter}$$

The above four energy terms can be simplified as $\Delta G_{sh}=0.53\sigma_{s}$, $\Delta G_{dil}=\frac{3}{2}E{\left(\frac{\Delta L}{L}\right)}^{2}V_{m}$, $\Delta G_{stor}=Gb^{2}\rho V_{m}$ and $\Delta G_{inter}=70.9$. Here, $\sigma_{s}$ is the yield strength and E is Young’s modulus of austenite, treated as a constant. $\frac{\Delta L}{L}$ is related to the difference between the thermal expansion coefficients of ferrite and austenite. $V_{m}$ is molar volume of iron atoms. G is the shear modulus, b is the Burgers vector and ρ is the dislocation density in the formed martensite. It was found that the dilatation strain energy ($\Delta G_{dil}$) induced by thermal expansion coefficient difference between the formed martensite and austenite plays a key role in the non-chemical driving forces. A complicated equation (Equation (7)) depending on the chemical compositions was given in the literature [26] and it was found that with this improved item, the accuracy of the martensite transformation start temperature prediction model was significantly improved [70]. In this work, it was found that the coefficient of linear thermal expansion of the pure metals corresponding to specific alloying elements was the most important feature influencing the MS temperature, as shown in Figure 6c. This coincidence indicated the rationality and superiority of contracting new features based on atomic parameters in the training of the phase transformation temperature prediction model.

(7) $$\frac{\Delta L}{L}=1.29+2.84X_{Ni}+1.45X_{C}-3.02X_{Cr}-10.7X_{Mn}-61.3X_{Si}-8.59\times 10^{-4}T-10.8x_{Ni}^{2}-270X_{Ni}X_{C}+3.38\times 10^{-10}X_{Ni}X_{Cr}-2\times 10^{-9}X_{Ni}X_{Mn}+\;1.83\times 10^{-9}X_{Ni}X_{Si}-1.16\times 10^{3}{X_{Ni}}^{T}-173x_{C}^{2}+1.68\times 10^{-11}X_{C}X_{Cr}+3.11\times 10^{-11}X_{C}X_{Mn}-9.95\times 10^{-13}X_{C}X_{Si}-8.03\times 10^{3}{X_{C}}^{T}+36x_{Cr}^{2}+\;0X_{Cr}X_{Mn}+0X_{Cr}X_{Si}-9.96\times 10^{-4}{X_{Cr}}^{T}+121x_{Mn}^{2}+0X_{Mn}X_{Si}-1.15\times 10^{-3}{X_{Mn}}^{T}+3.58\times 10^{3}x_{Si}^{2}+6.89\times 10^{-3}{X_{Si}}^{T}+1.48\times 10^{-8}T^{2}$$

### 4.2. The Influencing Factors of BS Temperature

**Figure 10:**
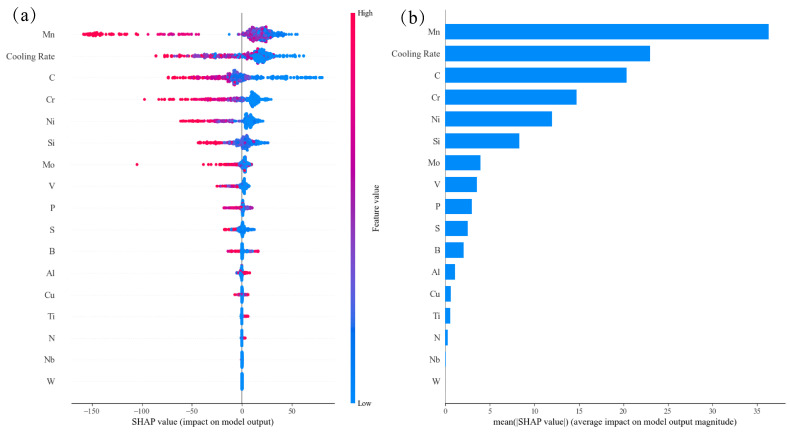
SHAP analysis in the trained BS prediction model: (**a**) impact on model output and (**b**) average impact on model output magnitude.

The incubation time was needed for bainite transformation as characterized by its time–temperature–transformation curves. Therefore, cooling rate outperformed other features in the feature importance ranking during the training, as shown in Figure 6d. Figure 10a clearly shows that the higher the C, Mn, Cr, Ni, Si, Mo, V, P and S contents and cooling rates, the lower the SHAP values (negative), i.e., the lower the BS temperature; the higher the Al, Ti and N contents, the higher the SHAP values (positive), i.e., the higher the BS temperature; Mn, Cu and B demonstrated more complicated effects on the MS temperature. The difference in the effects of alloying elements on the MS and BS temperatures indicated the diverse phase transformation mechanisms between martensite and bainite transformations. Meanwhile, the atomic Waber–Crome pseudopotential radius outperforms the atomic radius and the coefficient of linear thermal expansion in improving the performance of the BS prediction model, which indicated that the motion of the alloying element ion and the interaction between the alloying element ion and the surrounding electrons played an important role in bainite transformation and then the BS transformation could be better classified into reconstructive phase transformation.

**Figure 11:**
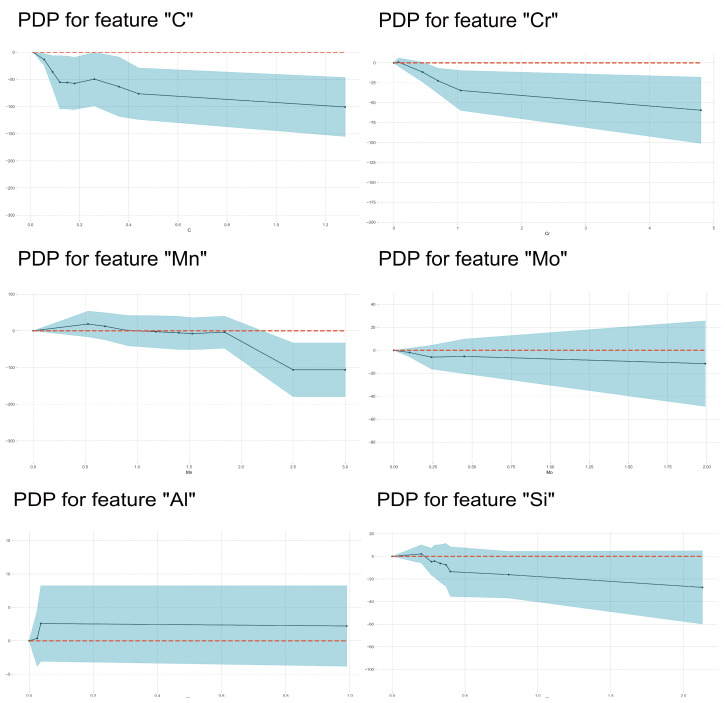
PDP analysis on the effects of alloying element content on BS temperature.

In Figure 11, it is demonstrated that the BS temperature firstly increased with the Mn and Si contents then decreased with increasing Mn and Si contents within the medium content range and finally increased again with further increasing Si content. It is also found that there was a clear plateau on most of the PDP curves, which were diverse from martensite transformation. The higher the carbon content, the lower the bainite transformation temperature.

It was reported that bainite transformation kinetics of Fe–C–Si–Mn alloys were much slower than those of ternary Fe–C–Mn and Fe–C–Si alloys, which suggested that the interaction of Si and Mn had an important influence on the kinetics of bainite transformation in Fe–C–Si–Mn alloys. It was experimentally proven that Si could enhance Mn segregation to austenite grain boundaries and inhibit the Fe_3_C precipitation and then inhibit the formation of bainitic ferrite nucleation [71]. For low-carbon bainite steels, it was found that Nb addition retarded bainitic transformation, and Mo addition was effective in promoting bainitic transformation [72], and the addition of B slightly decreased the bainite transformation temperature at low cooling rates, whereas the combined addition of B + Nb greatly decreased the transformation temperature [73]. Meanwhile, carbide-forming elements, such as Mo, Nb, V and Cr, lead to an elevation in the activation energy required for carbon diffusion in austenite, which then retards bainite formation [74].

**Figure 12:**
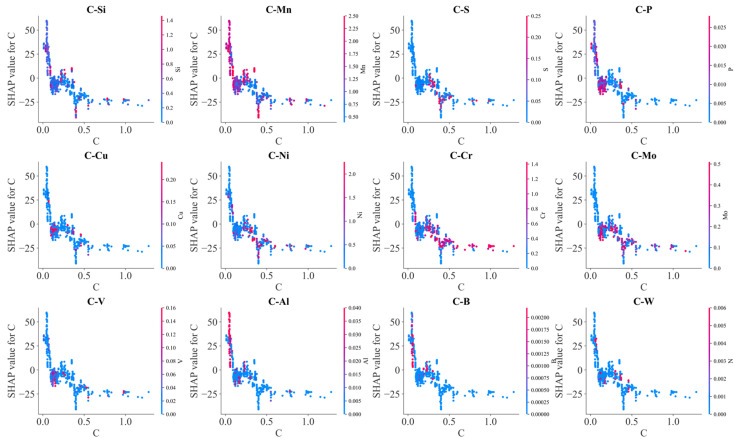
SHAP analysis on the effects of alloy element C on BS temperature.

Figure 12 illustrates the effects of the interaction between C and other alloying elements on SHAP values. Generally, SHAP values decreased with C content. However, due to the interaction with other alloying elements, scattering in SHAP values occurred at each carbon content. Mn, Mo, V, Al, Cr and B exhibited an obvious influence on the effects of carbon on BS temperature. At lower carbon contents, SHAP values of carbon increased with Mn, Al and B contents, and SHAP values of carbon decreased with increasing Mo and V contents at the higher carbon contents. Meanwhile, increasing S and P somehow decreased the SHAP values of carbon. These results indicated that Mn, Al and B decrease the lowering effects of C on the BS temperature, and Mo and V as well as S and P enhanced the lowering effects of C on the BS temperature. C–Cr interaction demonstrated a complicated influence on the BS temperature. At lower carbon content, C–Cr interaction decreased the BS temperature; at higher carbon content, C–Cr interaction increased the BS temperature. At high carbon content, C–Mn interaction tended to decrease the BS temperature. C–Si interaction slightly increased the BS temperature.

### 4.3. The Influencing Factors of Ac1 Temperature

**Figure 13:**
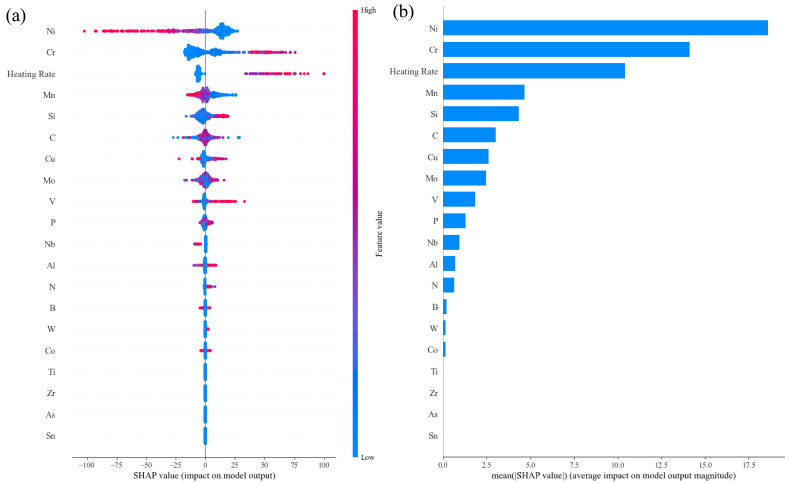
SHAP analysis in the trained Ac1 prediction model: (**a**) impact on model output and (**b**) average impact on model output magnitude.

Figure 13a demonstrates the importance of the features on the final prediction output in the trained models. The importance of the alloying elements was arranged in the following order, Ni, Cr, Mn, Si, C, Cu, Mo, V, P, Nb, Al, N, B, W, Co, Ti and Zr. Generally, Ac1 temperature decreased with increasing contents of austenite-forming elements, especially Ni and Mn, and increased with increasing contents of ferrite-forming elements, especially Cr and Si.

**Figure 14:**
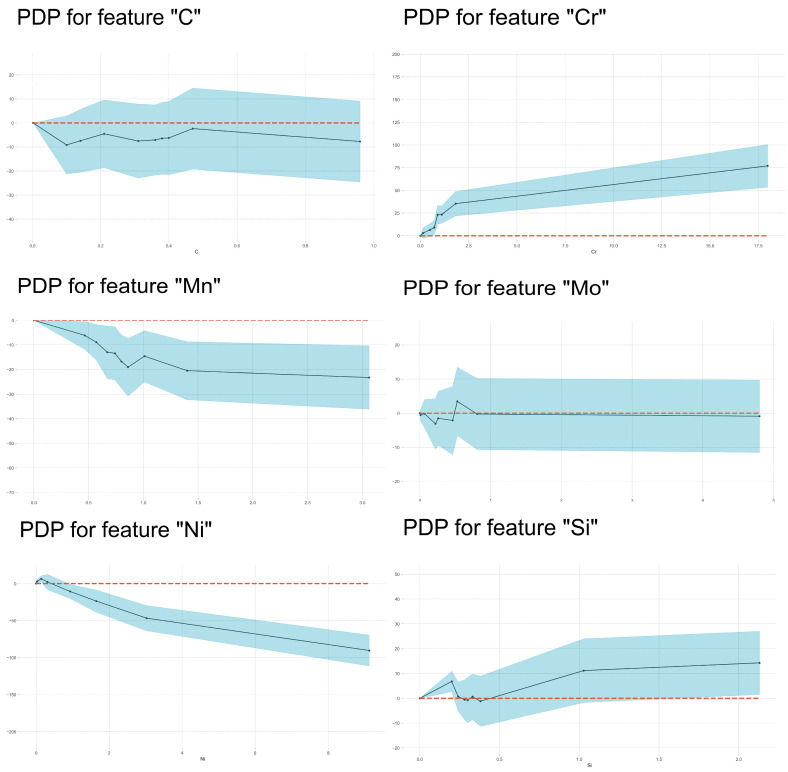
PDP analysis on the effects of alloying element content on Ac1 temperature.

Figure 14 further exhibits the dependence of Ac1 temperature on alloying element contents based on the PDP analysis. Unusually, it was found that at lower contents, the austenite-forming element Ni increased the Ac1 temperature, i.e., retarded austenite formation, and Si increased Ac1 temperature, i.e., prompted austenite formation. For example, there were two stages regarding the change in Ac1 temperatures of the investigated steels with the varying Si content. In the range of 0–1.0 wt% Si, the influence of Si content on the Ac1 temperatures was weak. And in some alloys, the influence of Ni content on the Ac1 temperatures was also weak. In the scope of this study, the effects of Ni and Si on Ac1 are summarized above. In another early work [75], 80 entries were used for training and 40 entries (randomly selected) were used for testing a trained network, including steels such as structural steels, stainless steels, rail steels, spring steels, high-temperature creep-resisting steels and tool steels. It was observed that Ac1 increased with Si content and decreased with increasing Ni content.

**Figure 15:**
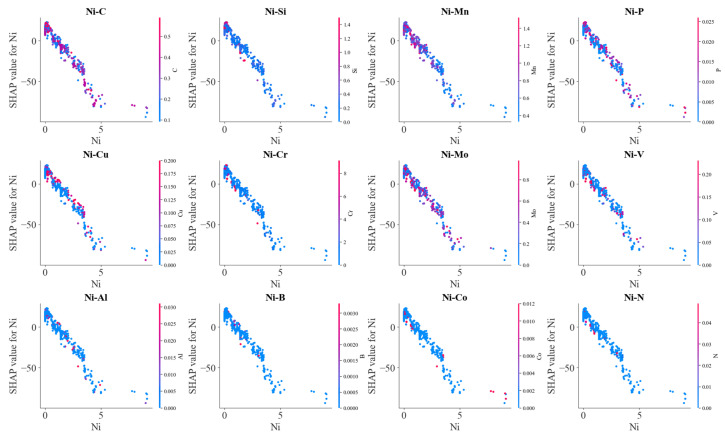
SHAP analysis on the effects of alloy element Ni on Ac1 temperature.

Mn and C always prompted austenite formation, and Cr inhibited austenite formation. However, the effect of C on Ac1 temperature remained constant after its content exceeded a certain amount. Generally, Ac1 temperature rises with increasing MP temperature (i.e., with a decrease in C content in the steel). Therefore, ferrite transformation in austenite should be inhibited. Figure 15 shows the influence of other alloying elements on the effects of Ni on Ac1 temperature. It was also found that at a lower concentration range, the interaction of Si, Cu and Cr with Ni could increase the SHAP values, i.e., Ac1 temperature. In other words, the ferrite-forming elements could weaken the effects of austenite-forming elements on Ac1 temperature.

### 4.4. The Influencing Factors of Ac3 Temperature

**Figure 16:**
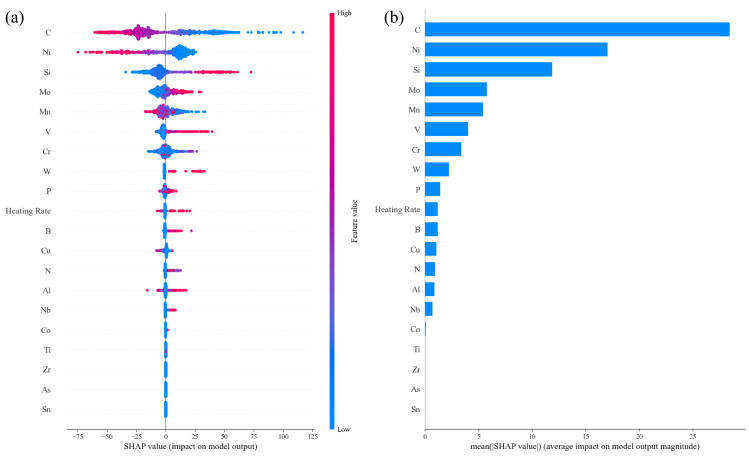
SHAP analysis in the trained Ac3 prediction model: (**a**) impact on model output and (**b**) average impact on model output magnitude.

**Figure 17:**
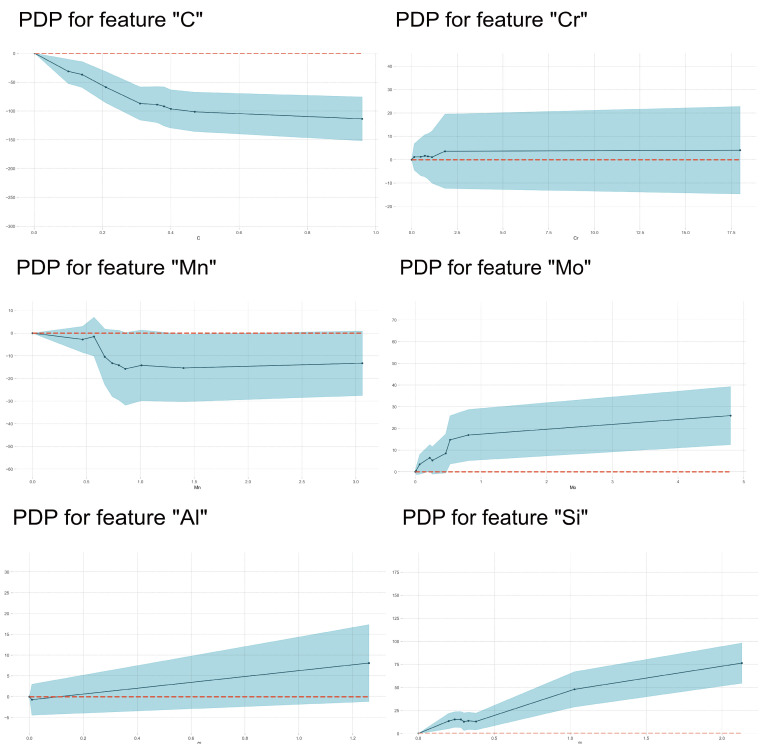
PDP analysis on the effects of alloying element content on Ac3 temperature.

In Figure 16, the importance of features in influencing Ac3 temperature is shown. It was clear that austenite-forming alloying elements and ferrite-forming elements were separated well by their SHAP values’ characteristics. But the span of SHAP values was smaller than those in the other three models, which indicated that the total effects of alloying elements were weaker. For all austenite-forming alloying elements, the Ac3 temperature decreased with increasing alloying content because these alloying elements expanded the area of the austenite phase and decreased the equilibrium Ac3 temperature. Meanwhile, Ac3 temperature increased with increasing ferrite-forming alloying element contents because these alloying elements expanded the area of the ferrite phase and increased the equilibrium Ac3 temperature. Therefore, the effects of alloying elements on Ac3 temperature mainly depended on their influence on the equilibrium phase boundary. However, Figure 17 shows that the effects of Mn on Ac3 temperature only increase with its content within a narrow range (about 0.6–0.8 wt%), which was consistent with the literature [20], where the authors found that an increase in Mn content had little effect on the Ac3 temperatures of their investigated steels. The C, Si and Mo elements demonstrated enhanced effects with increases in their contents. The effect of Mo on Ac3 temperatures was similar to that of Si but with lower magnitude. Si demonstrates the same influence on Ac1 and Ac3 temperatures as Al does.

**Figure 18:**
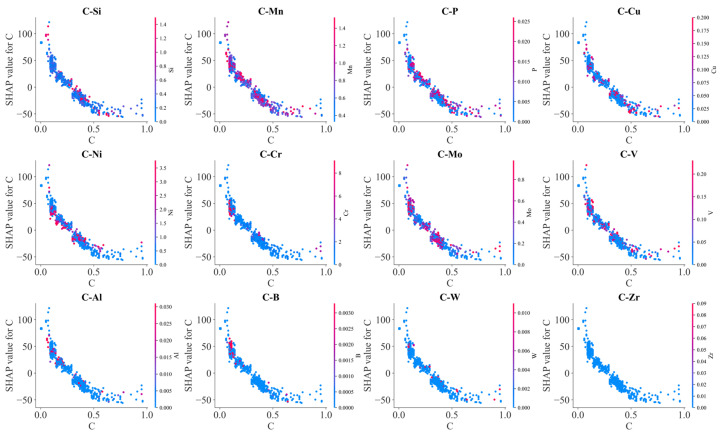
SHAP analysis on the effects of alloy element C on Ac3 temperature.

**Figure 19:**
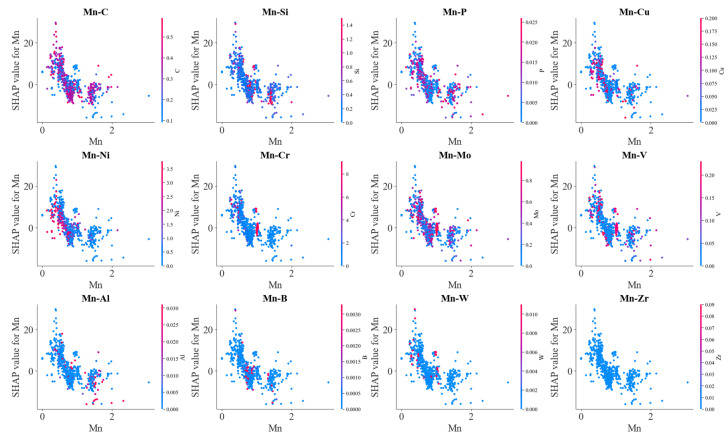
SHAP analysis on the effects of alloy element Mn on Ac3 temperature.

In Figure 18, it is shown that the interaction of C and alloying elements on Ac3 temperature was limited compared with their effects on other phase transformation temperatures. However, the interaction between C–Si and C–B decreased the effects of C in lowering Ac3 temperature, and the interaction between C–Ni and C–Cr enhanced the effects of C in lowering Ac3 temperature. To understand the effects of Mn on Ac3 temperature, the interaction of Mn and other alloying elements was further presented in Figure 19. It was found that the scattering degree of SHAP values of Mn was significantly larger than that of C SHAP values, which indicated that the effect of Mn on Ac3 temperature was obviously influenced by other alloying elements. C decreased the effects of Mn in lowering the Ac3 temperature at lower and higher Mn contents, but Ni enhanced the effect of Mn in lowering the Ac3 temperature at a medium content, so did the C. Meanwhile, Mo as well as V and Cr decreased the effect of Mn in lowering the Ac3 temperature, and Si and Al slightly enhanced the effect of Mn.

### 4.5. The Generalization Ability of the Trained Models

**Figure 20:**
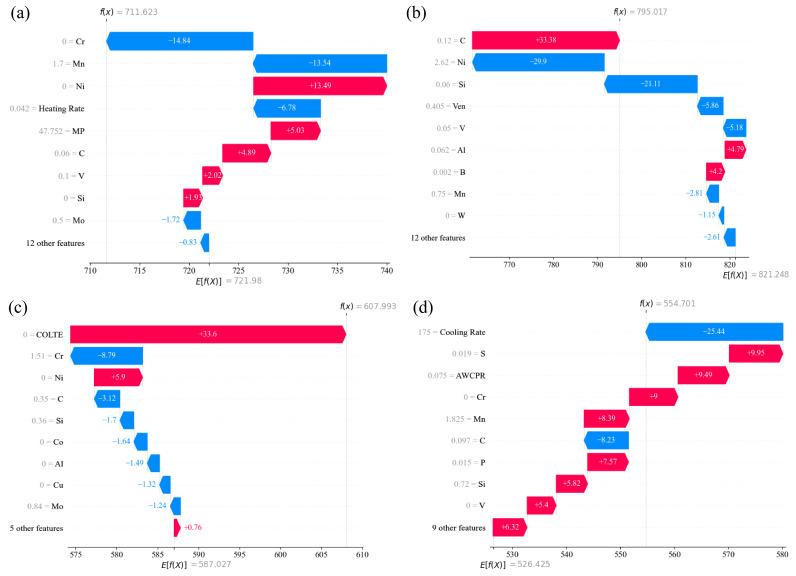
Examples of model predictions. (**a**) Ac1 (**b**) Ac3 (**c**) MS (**d**) BS.

The MAP dataset utilized in the present work had a large concentration range for all the main alloying elements. Therefore, it was expected the trained models showed good generalization ability on the unseen dataset. These best prediction models for each transformation temperature were chosen to evaluate their generalization ability on four groups of experimental phase transformation temperatures [6,56,61,76] not in the MAP project. Table 15, Table 16, Table 17 and Table 18 show comparisons between the experimental phase transformation temperatures and the predicted ones. It was generally found that the trained model with the best atomic parameter features gave better prediction, compared with the models without atomic parameter features. In Figure 20, the prediction process of the trained models is presented, where various features had distinct contributions to the final prediction. Table 16 demonstrates that the predicted MS temperatures were very close to the experimental ones. In the literature, Bohemen et al. [51] achieved the best results in the training of the MS temperature with MAE = 5.60, RMSE = 7.11, R2 = 0.98 and EV = 0.98 with a thermodynamics-based model including the effect of the prior austenite grain size. In our previous work [27], we achieved close prediction by considering the effects of alloying elements on the lattice constant of the prior austenite. In the present work, it was found that the coefficient of linear thermal expansion of the pure metals corresponding to specific alloying elements was the most important feature among 18 types of atomic features. Both features were related to the dilatation strain energy induced by austenite–martensite transformation, which contributed to most of the non-chemical driving force of the austenite–martensite phase transformation.

For bainite transformation start temperature prediction, it was found that the best model was created with MAE = 17.34, RMSE = 24.67 and R2 = 0.913, considering element characteristics in the form as shown in Table 19 [77]. In this work, it was found that atomic Waber–Crome pseudopotential radius comes first in the ranking of feature importance influencing the BS temperature. Considering atomic parameter-based features, the difference between the experimental and predicted BS temperatures was significantly narrowed with an averaged absolute error of 8.33, as shown in Table 16. However, the accuracy of BS temperature prediction was lower than that of the MS prediction model.

By using neural network to train the Ac1/Ac3 temperature prediction models, it is found that the absolute error value of predicted Ac1 temperature does not exceed 22 °C, and the relative error is less than 3.01%; the absolute error value of the predicted Ac3 temperature does not exceed 28 °C, and the relative error is less than 3.02% [75]. The results presented in Table 17 and Table 18 show that the prediction performance of the Ac1/Ac3 temperature prediction models trained in the present work by means of LightGBM was better, partially due to the quality of the MAP dataset and partially due to the improved LightGBM algorithm. For example, in earlier work, the averaged prediction errors of BS and MS temperatures were both larger than 20 °C using the trained artificial neural network model based on a dataset even with a narrowing of the chemical composition range, i.e., the total mass fractions of manganese, chromium, nickel and molybdenum did not exceed 5% [51], which indicated the importance of the data cleaning and feature engineering strategy and the advantage of newly developed machine learning algorithm. In the present work, considering the features based on atomic parameters, the prediction accuracy was significantly improved. Meanwhile, the performance of the Ac1 temperature prediction model was better than that of the Ac3 temperature prediction model, which was consistent with the literature [57].

## 5. Conclusions

Prediction models for MS, BS, Ac1 and Ac3 temperatures were trained using the popular machine learning algorithm LightGBM, considering new features constructed based on 18 atomic parameters. Most of the new features enhanced the performance of the trained model, and the underlying mechanisms were discussed in the perspective of phase transformation theories through PDP and SHAP analysis. The main conclusions could be drawn as follows:

(1) The prediction models for MS, BS, Ac1 and Ac3 temperatures were trained with high accuracy and achieved satisficed predictions on the unseen experimental data and exhibited higher accuracy and better generalization compared to the empirical formula. The prediction model for MS temperature showed the highest accuracy, followed by the Ac1 temperature prediction model.
(2) C, Ni and Cr are the top three elements influencing MS temperature, followed by Mn and Mo. MS temperature increased with increasing Al and Co contents. Other alloying elements exhibit positive or negative influences on MS temperature at different composition ranges.
(3) Except Al, Ti and N, the BS temperature generally decreased with increasing alloying element contents. Mn, Si and B elevated the BS temperature in certain content ranges.
(4) The averaged magnitude of the effects of alloying elements on phase transformation temperatures was highest for martensite transformation. Cooling rate and heating rate played important roles in bainite transformation during cooling and austenite transformation during heating, respectively.
(5) The interaction between alloying elements exhibits complicated effects on phase transformation temperatures. A linear relationship between the alloying element concentration and phase transformation temperature is hardly observed due to its contribution to both aspects, i.e., chemical driving forces and non-chemical driving forces as well as the interaction between alloying elements.

## Figures and Tables

**Table 1:** Dataset size after data cleaning.

Dataset	The Sample Size
MS	800
Ac1	735
Ac3	735
BS	655

**Table 2:** Number of deleted samples.

Dataset	Number
MS	150
Ac1	54
Ac3	53
BS	49

**Table 3:** Overall information of MS dataset.

Elements	Minimum	Maximum	Mean	Standard Deviation
Carbon (wt.%)	0.0016	1.8	0.36	0.247
Silicon (wt.%)	0	3.8	0.394	0.441
Manganese (wt.%)	0	10.24	0.867	0.801
Sulfur (wt.%)	0	0.054	0.003	0.007
Phosphorus (wt.%)	0	0.044	0.003	0.008
Cuprum (wt.%)	0	1.49	0.035	0.108
Nickel (wt.%)	0	31.3	2.026	5.023
Chromium (wt.%)	0	17.98	1.146	2.295
Molybdenum (wt.%)	0	8	0.305	0.587
Niobium (wt.%)	0	0.23	0.001	0.012
Vanadium (wt.%)	0	3.29	0.068	0.215
Titanium (wt.%)	0	1.613	0.003	0.058
Aluminum (wt.%)	0	3.006	0.019	0.151
Boron (wt.%)	0	0.006	0.00003	0.00003
Tungsten (wt.%)	0	18.59	0.192	1.334
Cobalt (wt.%)	0	30	0.233	1.821
Nitrogen (wt.%)	0	0.614	0.006	0.041
MS (K)	215.15	769	588.26	90.15

**Table 4:** Overall information of the Ac1 and Ac3 datasets.

Elements	Minimum	Maximum	Mean	Standard Deviation
Carbon (wt.%)	0	0.96	0.3	0.1661
Silicon (wt.%)	0	2.13	0.3859	0.4122
Manganese (wt.%)	0	3.06	0.8211	0.3833
Sulfur (wt.%)	0	0.09	0.0068	0.0106
Phosphorus (wt.%)	0	0.12	0.0079	0.012
Cuprum (wt.%)	0	2.01	0.0456	0.1275
Nickel (wt.%)	0	9.12	1.0069	1.4806
Chromium (wt.%)	0	17.98	1.2245	2.3783
Molybdenum (wt.%)	0	4.80	0.3215	0.3733
Niobium (wt.%)	0	0.17	0.0032	0.0128
Vanadium (wt.%)	0	2.45	0.0513	0.1324
Titanium (wt.%)	0	0.18	0.0014	0.014
Aluminum (wt.%)	0	1.26	0.0063	0.0604
Boron (wt.%)	0	0.05	0.0004	0.0029
Tungsten (wt.%)	0	8.59	0.0635	0.4791
Arsenic (wt.%)	0	0.019	0	0.0006
Stannum (wt.%)	0	0.008	0	0.0002
Zirconium (wt.%)	0	0.09	0.0001	0.0032
Cobalt (wt.%)	0	4.07	0.0615	0.4175
Nitrogen (wt.%)	0	0.06	0.0033	0.0118
Oxygen (wt.%)	0	0.005	0	0.0001
Heating Rate	0.027	50	1.0937	4.2398
Ac1 (K)	530	921	724.12	52.2347
Ac3 (K)	651	1060	819.83	55.1432

**Table 5:** Overall information of BS dataset.

Elements	Minimum	Maximum	Mean	Standard Deviation
Carbon (wt.%)	0.0114	1.28	0.231	0.193
Silicon (wt.%)	0	2.13	0.404	0.409
Manganese (wt.%)	0	3.00	1.308	0.666
Sulfur (wt.%)	0	2.13	0.057	0.249
Phosphorus (wt.%)	0	0.92	0.017	0.087
Cuprum (wt.%)	0	0.34	0.036	0.082
Nickel (wt.%)	0	5.25	0.425	0.849
Chromium (wt.%)	0	4.8	0.377	0.625
Molybdenum (wt.%)	0	1.99	0.121	0.213
Niobium (wt.%)	0	0.061	0.002	0.01
Vanadium (wt.%)	0	2.1	0.028	0.108
Titanium (wt.%)	0	0.14	0.004	0.017
Aluminum (wt.%)	0	0.99	0.01	0.041
Boron (wt.%)	0	0.003	0.0003	0.0007
Tungsten (wt.%)	0	18.59	0.038	0.743
Nitrogen (wt.%)	0	0.074	0.001	0.007
Cooling rate	0	790	127.33	161.48
BS (K)	149	780	527.44	87.2692

**Table 6:** Atomic parameter candidates utilized for constructing new features.

Abbreviation	Description
AR	Atomic radius
ACR	Atomic covalent radius
AMR	Atomic metallic radius
AWCPR	Atomic Waber–Crome pseudopotential radius
PE	Pauling electronegativity
PCS	Pettifor chemical scale
VEN	Valence electron numbers
MV	Molar volume, cm^3^
AN	Atomic number
MP	Melting point
DOS	Density of solid, kg/m^3^
TC	Thermal conductivity
COLTE	Coefficient of linear thermal expansion
EOF	Enthalpy of fusion
CR	Crystal Radius
MHC	Molar heat capacity
DFCE	Distance from core electron
DAVE	Distance from valence electron

**Table 7:** Empirical formulas for MS calculation.

No.	Ref.	Formulas
1	[21]	MS (°C) = 496 × (1 − 0.62C)×(1 − 0.092Mn) × (1 − 0.033Si) × (1 − 0.045Ni) × (1 − 0.07 Cr) × (1 − 0.029Mo) × (1 − 0.018W) × (1 − 0.012Co)
2	[21]	MS (°C) = 531 − 391.2C − 42.3Mn − 16.2Cr − 21.8Ni
3	[21]	MS (°C) = 565 − 600 × (1 − Exp(−0.96C)) − 31Mn − 12Si − 10Cr − 8Ni − 12Mo
4	[21]	MS (°C) = 520 − 320C − 50Mn − 30Cr − 20 × (Ni + Mo) − 5(Cu + Si)
5	[52]	MS (K) = 545 − 601.2 × (1 − (−0.868C)½) − 34.4Mn − 13.7Si − 9.2Cr − 17.3Ni − 15.4Mo + 10.8V + 4.7Co − 1.4Al − 16.3Cu − 361Nb − 2.44Ti − 3448B
6	[52]	MS (°C) = 692 − 37Mn − 14Si + 20Al − 11Cr − 502(C + 0.86N)½
7	[21]	MS (°C) = 538 − 350C − 37.7Mn − 37.7Cr − 18.9Ni − 27Mo
8	[21]	MS (°C) = 499 − 324C − 32.4Mn − 27Cr − 16.2Ni − 10.8 (Si + Mo + W)
9	[53]	MS (K) = 764.2 − 302.6C − 30.6Mn − 16.6Ni − 8.9Cr + 2.4Mo + 11.3Cu + 8.58Co + 7.4W − 14.5Si
10	[21]	MS (°C) = 499 − 292C − 34.2Mn − 10.8Si − 22Cr − 16.2Ni − 10.8Mo
11	[21]	MS (°C) = 539 − 423C − 30.4Mn − 7.5Si − 12.1Cr − 17.7Ni − 7.5Mo + 10Co
12	[21]	MS (°C) = 41.7 × (14.6 − Cr) + 61.1 × (8.9 − Ni) + 33.3 × (1.33 − Mn) + 27.8 × (0.47 − Si) + 1666.7 × (0.068 − C-N) − 17.8
13	[54]	MS (°C) = 541 − 401C − 36Mn − 10.5Si − 14Cr − 18Ni − 17Mo
14	[21]	MS (°C) = 499 − 308C − 32.4Mn − 27Cr − 16.2Ni − 10.8Si − 10.8Mo
15	[21]	MS (°C) = 561 − 474C − 33Mn − 17Cr − 17Ni − 21Mo

**Table 8:** Empirical formulas for Ac1 calculation.

No.	Ref.	Equations
1	[57]	Ac1 (°C) = 755.68 + 14.39Si − 26.86Mn + 16.32Cr − 18.5Ni + 88.91V
2	[54]	Ac1 (°C) = 742 − 29C − 14Mn + 13Si + 16Cr − 17Ni − 16Mo + 45V + 36Cu
3	[25]	Ac1 (°C) = 739 − 22C − 7Mn + 2Si + 14Cr − 13Ni − 13Mo − 20V
4	[55]	Ac1 (°C) = 723 − 10.7Mn − 6.9Ni + 29Si + 16.9Cr + 290As + 6.38W
5	[20]	Ac1 (°C) = 723 − 10.7Mn − 13.9Ni + 29Si + 16.9Cr + 290As + 6.38W

**Table 9:** Empirical formulas for Ac3 calculation.

No.	Ref.	Equations
1	[20]	Ac3 (°C) = 910 − 203C½ − 15.2Ni + 44.7Si + 104W + 31.5Mo + 13.1W
2	[55]	Ac3 (°C) = 910 − 203C − 15.2Ni + 44.7Si + 104W + 31.5Mo + 13.1W − (30Mn + 11Cr + 20Cu − 700P − 400Al − 120As − 400Ti)
3	[58]	Ac3 (°C) = 902 − 255C − 11Mn + 19Si − 20Ni − 5Cr + 13Mo + 55V
4	[57]	Ac3 (°C) = 928 − 236.37C + 30.44Si − 32.68Mn − 27.51Ni + 141.65V
5	[54]	Ac3 (°C) = 925 − 219C½ − 7Mn + 39Si − 16Ni + 13Mo + 97V

**Table 10:** Empirical formulas for BS calculation.

No.	Ref.	Equations
1	[22]	BS (°C) = 711 − 362C + 262C^2^ − 28Mn + 44Si
2	[56]	BS (°C) = 720 − 585.63C + 126.6C^2^ − 66.34Ni + 6.06Ni^2^ − 0.232Ni^3^ − 31.66Cr + 2.17Cr^2^ − 91.68Mn + 7.82Mn^2^ − 0.3378Mn^3^ − 43.37Mo + 9.16Co − 0.1255Co^2^ + 0.000284Co^3^ − 36.02Cu − 46.15Ru
3	[56]	BS (°C) = 844 − 597C − 63Mn − 16Ni − 78Cr
4	[56]	BS (°C) = 830 − 270C − 90Mn − 37Ni − 70Cr − 86Mo
5	[56]	BS (°C) = 630 − 45Mn − 40V − 35Si − 30Cr − 258Mo − 20Ni − 15W

**Table 11:** Feature sets of machine learning models.

Phase Transformation Temperature	Feature Set
Ac1	C, Si, Mn, S, P, Cu, Ni, Cr, Mo, Nb, V, Ti, Al, B, W, As, Sn, Zr, Co, N, Heating Rate
Ac3	C, Si, Mn, S, P, Cu, Ni, Cr, Mo, Nb, V, Ti, Al, B, W, As, Sn, Zr, Co, N, Heating Rate
MS	C, Si, Mn, S, P, Cu, Ni, Cr, Mo, Nb, V, Ti, Al, B, W, Co, N
BS	C, Si, Mn, S, P, Cu, Ni, Cr, Mo, Nb, V, Ti, Al, B, W, N, Cooling Rate

**Table 12:** Model performance before adding atomic parameters.

	MAE	R2	TrainScore	TestScore
Ac1	11.092	0.950	0.995	0.950
Ac3	10.149	0.920	0.993	0.920
MS	12.058	0.942	0.987	0.942
BS	18.622	0.928	0.978	0.928

**Table 13:** Model performance after adding atomic parameters.

	MAE	R2	TrainScore	TestScore
Ac1	9.488	0.960	0.996	0.960
Ac3	9.217	0.939	0.995	0.939
MS	7.273	0.984	0.993	0.984
BS	15.963	0.943	0.981	0.943

**Table 14:** Clarification of the normal alloying elements in the steels.

Austenite-forming elements	C, N, Cu, Mn, Ni, Co
Ferrite-forming elements	Cr, V, Si, Al, Ti, Mg, W
Carbide-forming elements	Ti, Zr, V, Ta, Nb, W, Mo, Cr, Mn
Non-carbide-forming elements	Ni, Co, Si, Al, Cu

**Table 15:** Verification of MS temperature prediction model.

Steels	Experiment	Prediction (without Atomic Parameters)	Prediction (with Atomic Parameters)
En325	390 °C	395 °C (+5.0 °C)	391 °C (+1.0 °C)
En11	280 °C	276 °C (−4.0 °C)	279 °C (−1.0 °C)
En17	315 °C	320 °C (+5.0 °C)	317 °C (+2.0 °C)
En23	310 °C	313 °C (+3.0 °C)	310 °C (+0.0 °C)
En320	415 °C	429 °C (+14 °C)	425 °C (+10 °C)

**Table 16:** Verification of BS temperature prediction model.

Steels	Experiment	Prediction (without Atomic Parameters)	Prediction (with Atomic Parameters)
No.64	495 °C	488 °C (−7.0 °C)	493 °C (−2.0 °C)
No.38	430 °C	408 °C (−22 °C)	412 °C (−18 °C)
No.36	520 °C	531 °C (+11 °C)	525 °C (+5.0 °C)

**Table 17:** Verification of Ac1 temperature prediction model.

Steels	Experiment	Prediction (without Atomic Parameters)	Prediction (with Atomic Parameters)
1#	810 °C	816 °C (+6.0 °C)	809 °C (−1.0 °C)
2#	785 °C	799 °C (+14 °C)	792 °C (+7.0 °C)
3#	825 °C	831 °C (+6.0 °C)	826 °C (+1.0 °C)
4#	820 °C	829 °C (+9.0 °C)	821 °C (+1.0 °C)
5#	808 °C	825 °C (+17 °C)	820 °C (+12 °C)

**Table 18:** Verification of Ac3 temperature prediction model.

Steels	Experiment	Prediction (without Atomic Parameters)	Prediction (with Atomic Parameters)
0.03C-0.076N	920 °C	938 °C (+18 °C)	930 °C (+10 °C)
0.05C-0.024N	950 °C	928 °C (−22 °C)	936 °C (−14 °C)
0.05C-0.037N	950 °C	926 °C (−24 °C)	935 °C (−15 °C)
0.05C-2W	950 °C	924 °C (−26 °C)	935 °C (−15 °C)
0.07C-2W	930 °C	927 °C (−3.0 °C)	929 °C (−1.0 °C)

**Table 19:** Features related to the atomic parameters.

Feature	Feature Description	Formula
Sum_Atom_R	Summation of atomic radius	$\sum_{i=1}^{17}a_{i}r_{i}$
Atom_diff(Fe)	Atomic radius difference (Take Fe as reference)	∑i=117ai(1−rirFe)2
Sum_VEN	Total valence electron number	$\sum_{i=1}^{17}a_{i}VEC_{i}$
VEN_Fe	Valence electron number difference (Take Fe as reference)	∑i=117ai(1−VECiVECFe)2
VEN_C	Valence electron number difference (Take C as reference)	∑i=117ai(1−VECiVECC)2
Sum_EN	Pauling electronegativity	$\sum_{i=1}^{17}a_{i}\chi_{i}$
EN_ Fe	Electronegativity difference (Take Fe as reference)	∑i=117ai(1−χiχFe)2
EN_C	Electronegativity difference (Take C as reference)	∑i=117ai(1−χiχC)2

Note: *a_i_* is the mole fraction of the alloying element, *r_i_* is the atomic radius of the alloying element or ion, *VEC_i_* is the number of valence electrons of the alloying element or ion, $\chi_{i}$ is the electronegativity of the alloying element or ion.

## Data Availability

The data that support the findings of this study are available from the corresponding authors upon reasonable request.
